# Incidence and Risk Factors of Delirium in the Intensive Care Unit: A Prospective Cohort

**DOI:** 10.1155/2021/6219678

**Published:** 2021-01-08

**Authors:** Farshid Rahimi-Bashar, Ghazal Abolhasani, Nahid Manouchehrian, Nasrin Jiryaee, Amir Vahedian-Azimi, Amirhossein Sahebkar

**Affiliations:** ^1^Department of Anesthesiology and Critical Care, School of Medicine, Hamadan University of Medical Sciences, Hamadan, Iran; ^2^Department of Anesthesiology, Fatemi Medical Center, Hamadan University of Medical Sciences, Hamadan, Iran; ^3^Department of Community Medicine, Faculty of Medicine, Hamadan University of Medical Sciences, Hamadan, Iran; ^4^Trauma Research Center, Nursing Faculty, Baqiyatallah University of Medical Sciences, Tehran, Iran; ^5^Biotechnology Research Center, Pharmaceutical Technology Institute, Mashhad University of Medical Sciences, Mashhad, Iran; ^6^Neurogenic Inflammation Research Center, Mashhad University of Medical Sciences, Mashhad, Iran; ^7^Halal Research Center of IRI, FDA, Tehran, Iran; ^8^School of Pharmacy, Mashhad University of Medical Sciences, Mashhad, Iran

## Abstract

**Purpose:**

The purpose of this study was to determine the incidence, risk factors, and impact of delirium on outcomes in ICU patients. In addition, the scoring systems were measured consecutively to characterize how these scores changed with time in patients with and without delirium. *Material and Methods*. A prospective cohort study enrolling 400 consecutive patients admitted to the ICU between 2018 and 2019 due to trauma or surgery. Patients were followed up for the development of delirium over ICU days using the Confusion Assessment Method (CAM) for the ICU and Intensive Care Delirium Screening Checklist (ICDSC). Cox model logistic regression analysis was used to explore delirium risk factors.

**Results:**

Delirium occurred in 108 (27%) patients during their ICU stay, and the median onset of delirium was 4 (IQR 3–4) days after admission. According to multivariate cox regression, the expected hazard for delirium was 1.523 times higher in patients who used mechanical ventilator as compared to those who did not (HR: 1.523, 95% CI: 1.197-2.388, *P* < 0.001).

**Conclusion:**

Our findings suggest that an important opportunity for improving the care of critically ill patients may be the determination of modifiable risk factors for delirium in the ICU. In addition, the scoring systems (APACHE IV, SOFA, and RASS) are useful for the prediction of delirium in critically ill patients.

## 1. Introduction

Delirium is an acute confusion that is associated with impaired consciousness, decline in cognitive function and attention, sudden onset, and a period of fluctuations [1]. It is associated with a rapid decline in brain function and is usually caused by diseases with systemic involvement [2]. It is clinically important because it has a considerable impact on morbidity and mortality rate (odds ratio: 1.95) and prolongs the length of hospitalization (added 2.2 day per admission) and the risk of permanent cognitive decline as morbidity [3, 4]. The incidence of delirium varies in the general population, elderly people, those with preexisting cognitive impairments and those who are admitted to an intensive care unit (ICU), from 2% to 80%. Therefore, recognizing the risk factors, prevention, and early treatment for delirium is important in patient care [5, 6].

The risk of delirium is dependent on a complex interplay between predisposing (patient-related) and precipitating (hospital-related) risk factors [7–10]. Predisposing factors include age, gender, comorbidities, and illness severity. Precipitating factors include medications (including sedatives), application of mechanical ventilation (MV), and hospital or ICU length of stay (LOS) [11–14]. Predictive scoring systems are widely used in ICUs to estimate the severity of illness and estimate the risk of mortality or to identify patients at high risk of dying [15–17]. Prior studies have shown that Acute Physiology and Chronic Health Evaluation (APACHE) II and Sequential Organ Failure Assessment (SOFA) scores in the ICUs are helpful and that trends in scores over time can predict the mortality dependent of initial score on admission [18–21]. Since the strong association between delirium and mortality, there has been no in-depth analysis focusing on sequential measurements of scoring systems to characterize how these scores changed with time and provide prognostic information among delirium patients in ICUs.

The purpose of this prospective study was to determine the incidence, risk factors, and impact of delirium on outcomes in ICU patients. In addition, the scoring systems were measured consecutively to characterize how these scores changed with time in patients with and without delirium.

## 2. Material and Methods

### 2.1. Study Design and Participants

In this prospective cohort study, 400 consecutive patients due to trauma or surgery admitted to the medical ICU at Be'sat Hospital in Hamadan, Iran, between 2018 and 2019 were screened for delirium. This cohort study was conducted and reported in accordance with the recommendations of the Strengthening the Reporting of Observational Studies in Epidemiology (STROBE) statement [22]. The study protocol was reviewed and approved by the Ethics Committee of Hamadan University of Medical Sciences, Hamadan, Iran, with code IR.UMSHA.REC.1397.861. In addition, patients or their relatives were informed about participation in the study by the physician at the time of admission with consent in all cases.

### 2.2. Inclusion and Exclusion Criteria

All over 18 years old patients, who are hospitalized for longer than 24 hours on the ICU due to trauma or surgery in the period from 2018 to 2019, were included in this study, except in the case of neurological illness (psychiatric illness or cognitive disorders), with deep sedation (Ramsay Sedation Scale (RSS) > 3 or Richmond Agitation Sedation Scale (RASS) ≤ –3), and severe brain injuries (Glasgow Coma Scale (GCS) ≤ 8), and if delirium assessment was impossible for patient.

### 2.3. Delirium Assessment

Delirium was assessed during each shift (three times daily; morning, noon, and evening), by trained nurses, using the Confusion Assessment Method for the ICU (CAM-ICU) [23] and Intensive Care Delirium Screening Checklist (ICDSC) screening tools [24]. The CAM-ICU assessment was positive if patients demonstrated an acute change or fluctuation in the course of their mental status (as determined by abnormalities), plus inattention, and either disorganized thinking or an altered level of consciousness. In terms of ICDSC, the checklist is scored out of eight categories that include the level of consciousness, inattention, disorientation, delusions, psychomotor agitation, inappropriate speech or mood, sleep disturbance, and symptom fluctuation [25]. Each category is coded as present (i.e., a score of 1) if the patient meets the criteria listed, for a maximum score of 8. Patients who scored ≥4 on the ICDSC at any time during the ICU stay were categorized as ever having delirium, and those with all of their ICDSC scores < 4 on all ratings were categorized as never having delirium.

### 2.4. Data Collection

A trained intensivist assigned to data collection (RB-F), collected demographic data (age, gender, marital status and smoking status based on their medical records), cause of ICU admission (trauma, nontrauma), usage of mechanical ventilator (MV), incidence of ventilator-associated pneumonia (VAP), cumulative dose of sedative, and analgesic medications and antibiotic therapy for all eligible patients in ICU. The diagnosis of VAP was performed based on the clinical pulmonary infection score (CPIS) [26]. Patients were monitored for 72 hours from admission and examined by an infection disease specialist. Bacterial pneumonia index calculated based on persistent infiltration in the chest X-ray, body temperature, white blood cell count, airways discharges, ratio of arterial blood oxygen to inhaled oxygen, and culture and smear of lung discharges. A patient was considered to have pneumonia if the score was more than 6 [27].

The severity of illness based on APACHE IV, SOFA, and RASS scores was calculated on admission and 14 days continuously and on day 28 for each patient. The APACHE IV tool uses variables derived from the worst values from the initial 24 hours of ICU admission [28, 29]. The SOFA score was initially designed to sequentially assess the severity of organ dysfunction in critically ill patients, and scores are calculated 24 hours after ICU admission [30]. Additionally, the RASS was administered four-hourly to assess sedation and patients with a RASS between –2 and +3 were administered the CAM-ICU and ICDSC to test for the presence of delirium [31, 32]. If delirium base occurred at any point during the 24 hours of the day, it was considered a day with delirium. Time to onset of ICU delirium was calculated from the patient's physician arrival in the ICU.

### 2.5. Outcomes

The primary outcome variables included mortality, hospital length of stay, and length of stay in the ICU period. In addition, we included 2 secondary outcome variables: mechanical ventilator uses and VAP during ICU stay.

### 2.6. Statistical Analysis

Data were expressed as the mean ± SD for continuous variables or median (interquartile range) for categorical characteristics. Patients' baseline demographic, clinical characteristics, and outcomes differences between patients with and without delirium were assessed using a *t*-test for continuous variables, and *χ*^2^ test was used for comparing categorical proportions. For the analysis of sedative and analgesic medications (morphine, methadone, fentanyl, midazolam, dexamethasone, diazepam, and hydrocortisone), the mean cumulative dose administered per patient during the ICU stay was used as summary measures and *t*-tests were used to compare distributions of the drugs between the no delirium and delirium groups. Univariate and multivariate logistic regression was employed to estimate the odds ratio (OR) to investigate the independence of risk factors for delirium. In addition, univariate and multivariate proportional hazard Cox regression, with delirium as the event and the time to onset of ICU delirium, was applied to analyze the relationship between time to delirium and risk factors. In the multivariate analyses, the significant variables in a backward selection modeling were reported as hazard ratio (HR) with 95% CI. To evaluate changes in predicting score systems such as APACHE IV, SOFA, and RASS over time, repeated measures analysis of variance (RMANOVA) was applied after adjusting for demographic baseline differences between patients with and without delirium. Also, pairwise comparisons were done by Bonferroni post hoc test. The assumption of sphericity was addressed by Mauchly's test of sphericity, and when the assumption was not satisfied (<0.05), the Greenhouse-Geisser correction of *P* value was utilized. All data were analyzed using the Statistical Package for the Social Sciences (SPSS) 21.0 statistical package (Chicago, IL, USA), and two-sided *P* < 0.05 indicated a statistically significant difference.

## 3. Results

### 3.1. Baseline Demographic, Clinical Characteristics, and Outcomes

Delirium occurred in 108 (27%) patients during their ICU stay, and the median onset of delirium was 4 (IQR 3–4) days after admission. Patients' baseline demographic, clinical characteristics, and outcomes between patients with and without delirium are presented in Table 1. There was no significant difference in age or cause of ICU admission in respect of trauma or nontrauma between delirious and nondelirious patients (*P* > 0.05). However, patients with delirium were mostly male, single, and smokers. Delirious patients significantly used higher mechanical ventilators, higher incidence of head trauma, and lower incidence of VAP and received more antibiotic therapy compared to patients without delirium. In terms of scoring system to predict the severity of illness, the mean scores of APACHE IV, SOFA, and RASS were significantly elevated in patients with delirium. In addition, the mean days of ICU and hospital LOS were significantly higher than that in nondelirious patients. In addition, mortality was significantly higher in the delirium group (*P* < 0.001).

### 3.2. Logistic Regression

In multivariate logistic regression analysis, unmarried status, smoking, use of mechanical ventilator, head trauma, high SOFA score, and high ICU LOS were independent risk factors for delirium. Univariate and multivariate logistic regression analyses to determine the independent factors associated with delirium are presented in Table 2.

### 3.3. Cox Regression

A proportional hazard Cox regression analysis with time-varying covariates, taking delirium as the event, and the time to onset of ICU delirium was used in the study, which are listed in Table 3. According to multivariate cox regression, the expected hazard for delirium is 1.523 times higher in patients who used mechanical ventilator as compared to those who did not (hazard ratio (HR): 1.523, 95% CI: 1.197-2.388, *P* < 0.001).

### 3.4. Time Trend of Severity of Illness Scoring Systems

**Figure 1:**
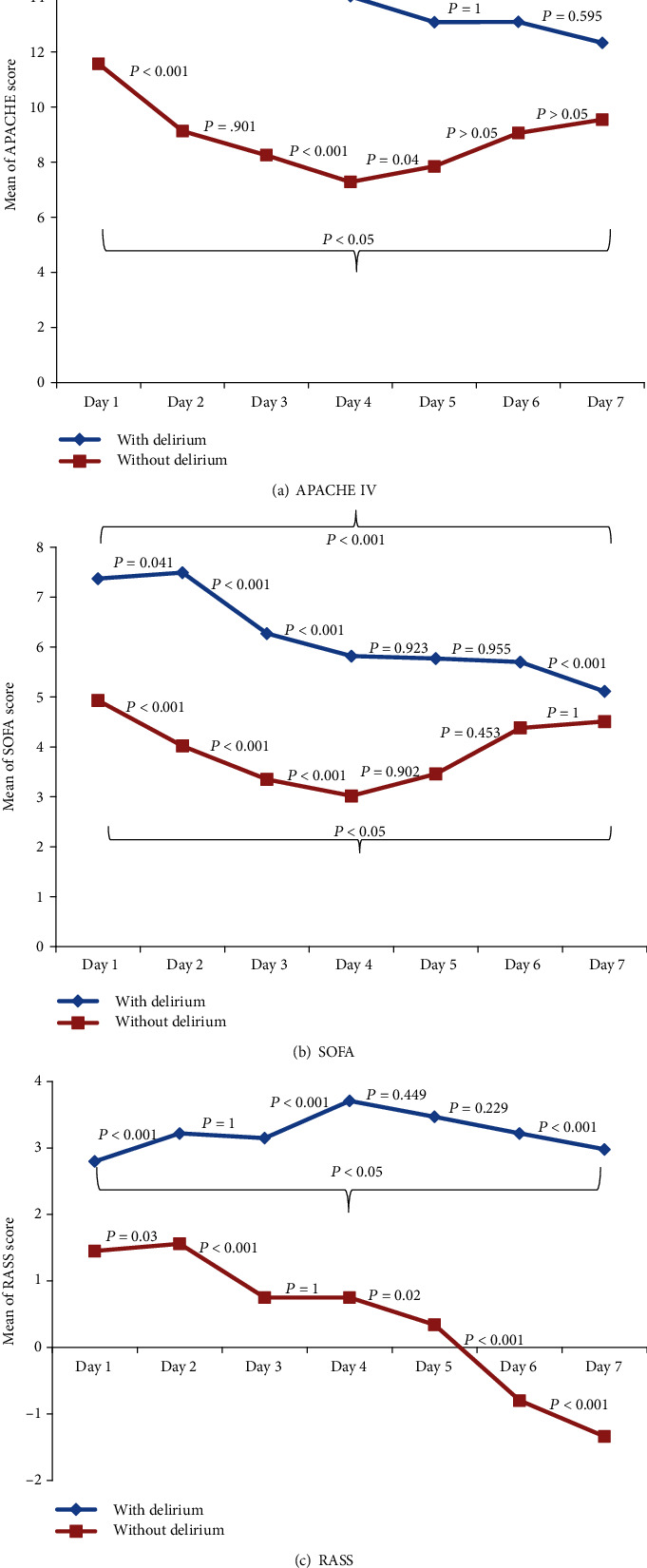
Plot of average scores for patients with and without delirium with respect to time. *P* values reflect pair-wise comparisons between consecutive time intervals, after adjusting for age, gender, marital status, and smoking status.

To characterize how severity of illness scoring systems changed relative to time, plots of average scores for 7 days from admission time in patients with and without delirium are shown in Figure 1. For each model, average scores of patients with delirium were higher than average scores of patients without delirium at everyday interval (*P* < 0.001). For the RASS score, the average score of patients with delirium was increased closer to time of onset of delirium (*P* < 0.001), while, in both APACHE IV and SOFA scores, we had a significant downward trend. In addition, time trends of scoring system for 14 days continuously and day 28 during ICU stay were recorded, which can see the results in Supplementary Table 1.

### 3.5. Sedative and Analgesic Medications

The mean cumulative administered dose of sedative and analgesic medication such as morphine, methadone, fentanyl, midazolam, dexamethasone, diazepam, and hydrocortisone used in this cohort is presented in Table 4. The mean cumulative doses of these medications were significantly higher in patients in the delirium group (*P* < 0.001), but only hydrocortisone was used in patients without delirium.

### 3.6. Antibiotic Therapy

**Figure 2:**
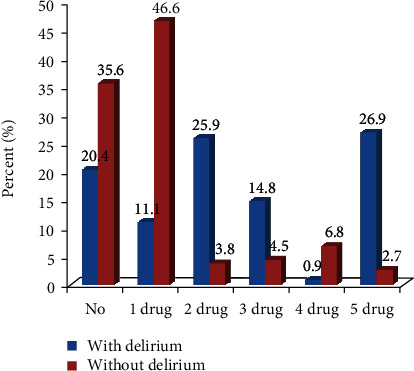
The status of antibiotic therapy in patients with and without delirium; one drug means single therapy with one type of antibiotic, and the others show combination therapy with 2, 3, 4, or 5 types of antibiotics.

Antibiotic therapy was given to 79.6% of the patients with delirium and to 64.4% of the patients without delirium. Patients with delirium received antibiotic significantly more often than patients without delirium (79.6% vs. 64.4%, respectively, *P* = 0.004). From 188 patients without delirium and 86 patients with delirium, who received antibiotic therapy, 136 (72.3%) and 12 (13.9%) patients had single therapy, respectively. However, 52 (27.6%) patients without delirium and 74 (86.04%) patients with delirium had combination therapy of antibiotics. The status of antibiotic therapy in patients with and without delirium is shown in Figure 2.

## 4. Discussion

Monitoring for delirium among critically ill patients in ICUs could allow the medical team to consider causes and modifications in their treatment of the patient experiencing this organ dysfunction [33]. The incidence of delirium in our study was 27% with the median onset of delirium 4 (IQR 3–4) days after admission and is comparable with previous studies in which incidences of 9%–40% have been reported in ICU patients [34–36]. However, it is much lower than the incidence of up to 80% presented by other authors [3, 37, 38]. The results of this prospective cohort study showed that patients with delirium were mostly male, single, and smokers. Moreover, our results confirm previous reports with respect to the association of delirium with elevated APACHE IV, SOFA, RASS scores, MV uses, longer stay in the ICU and hospital, and higher mortality [18, 39, 40]. All of these underlines again the importance of preventing delirium or at least the value of early diagnosis and treatment.

Interestingly, despite the increased use of MV in our patients with delirium, the rate of VAP in these patients was lower than in patients without delirium. This could be related to the implementation of antibiotic therapy in these patients that this hypothesis is consistent with a study conducted by Dale et al. [41]. In the present study, Acinetobacter species (38%) was found as a causative agent of VAP and then followed by Pseudomonas (27.5%), Staphylococcus aureus (13.8%), Klebsiella (10.3%), and Escherichia coli (10.3%). The half of (44.8%) microorganisms isolated as pathogens of VAP in this study were multidrug resistant (MDR). The results showed that patients with delirium were treated more with antibiotics than patients without delirium, which could lead to the reduced rate of VAP in the delirium group. Similar to our results, prior studies have reported that the clinical management of VAP depends on appropriate antimicrobial therapy, which needs to be selected based on individual patient factors and bacterial pathogens and antibiotic resistance patterns [42, 43].

In studying the scoring systems over time, we found that for the APACHE IV, SOFA, and RASS models average scores decreased significantly over time in both groups (with and without delirium). However, the mean scores were significantly higher in patients with delirium than in those without delirium. Elevated scores in the delirium group is a warning and can provide the physician sufficient time to reassess patients to the early identification of at-risk patients for delirium and also allows time for intervention measures to take effect and improve outcomes. These findings confirmed that these scores are useful for the prediction of delirium in critically ill patients. The downward trend of these scores in both groups of patients showed the positive effect of acting sedative and analgesic medications which can reduce the severity of illness. However, the progression of delirium was of clinical relevance above and beyond that attributed to the administration of sedative and analgesic medications.

The strengths of this research include the large number of patients enrolled (*n* = 400), and the study design was prospectively. All data were derived from daily monitoring (three times daily) of sedation scoring and delirium assessments by the bedside nurses as part of a multidisciplinary approach to care within the ICU using well-validated tools (RASS, CAM-ICU and ICDSC). Some previous studies on the incidence of delirium used 24-hour or weekly assessments, in which an estimated 60% to 80% of delirium was missed in the absence of daily monitoring [44, 45]. In fact, in addition to the logistic regression, we chose the more sophisticated approach using time-to-event analysis with Cox regression and the delirium as time-dependent covariates. Moreover, we assessed scores sequentially over time in delirious and non-delirious patients.

This study is not without limitations. First, in cohort studies, there might be unknown covariates that influence outcomes and it is not possible to match patients. Second, this observational investigation was not designed to prove the causal relationship between delirium and clinical outcomes. Therefore, we could not match the patients in terms of demographic characteristics, though our multivariable analysis was specifically designed to take these covariates into account. Thus, further evidence from randomized, prospective clinical trials will be helpful to confirm such a relationship.

## 5. Conclusion

Despite sedative and analgesic medications and antibiotic therapy, the development of delirium in ICU was associated with a 4-fold increase in the risk of death. Our findings suggest that an important opportunity for improving the care of critically ill patients may be the determination of modifiable risk factors for delirium in the ICU setting. Numerous risk factors for delirium have been identified by logistic regression including male gender, smoking, mechanical ventilation, head trauma, and prolonged ICU stay. However, time-to-event analysis with Cox regression showed mechanical ventilation use as the only independent risk factor for delirium. In addition, according to our results, the scoring systems (APACHE IV, SOFA, and RASS) are useful for the prediction of delirium in critically ill patients.

## Figures and Tables

**Table 1:** Baseline characteristics and outcomes of the participants according to with and without delirium.

Variables	Patients without delirium (*n* = 292)	Patients with delirium (*n* = 108)	Total patients (*n* = 400)	*P* value
Age, mean ± SD (years)	38.72 ± 13.58	39.87 ± 12.83	39.04 ± 13.37	0.446
Gender, male (%)	172 (58.9)	84 (77.8)	256 (64)	<0.001
Marital status, unmarried (%)	77 (26.4)	56 (51.9)	133 (33.2)	<0.001
Smoking, yes (%)	158 (54.1)	79 (73.1)	237 (59.3)	0.001
Cause of ICU admission, trauma (%)	223 (76.4)	79 (73.1)	302 (75.5)	0.506
Head trauma, yes (%)	43 (14.7)	71 (65.7)	114 (28.5)	<0.001
Usage of MV, yes (%)	109 (37.3)	87 (80.6)	196 (49)	<0.001
APACHE IV score, mean ± SD	11.57 ± 4.11	16.08 ± 3.84	12.79 ± 4.50	<0.001
SOFA score, mean ± SD	4.93 ± 1.70	7.37 ± 1.17	5.59 ± 1.91	<0.001
RASS, mean ± SD	1.48 ± 0.50	2.83 ± 0.81	1.85 ± 0.84	<0.001
VAP, yes (%)	42/109 (38.5)	16/87 (18.4)	58/196 (29.6)	0.006
Antibiotics therapy, yes (%)	188 (64.4)	86 (79.6)	274 (68.5)	0.004
Hospital LOS, mean ± SD (day)	10.48 ± 2.92	19.55 ± 4.57	12.93 ± 5.29	<0.001
ICU LOS, mean ± SD (day)	5.82 ± 1.97	13.66 ± 3.47	7.94 ± 4.26	<0.001
Outcome, mortality (%)	18 (6.2)	23 (21.3)	41 (10.2)	<0.001

APACHE IV: Acute Physiology and Chronic Health Evaluation IV; SOFA: Sequential Organ Failure Assessment; RASS: Richmond Agitation-Sedation Scale; MV: mechanical ventilator; VAP: ventilator-associated pneumonia; LOS: length of stay.

**Table 2:** Univariate and multivariate logistic regression analysis to determine the independent factors associated with delirium.

Variables	Univariate		Multivariate	
	OR (95% CI)	*P* value	OR (95% CI)	*P* value
Age	1.006 (0.990-1.023)	0.445		
Gender (male vs. female)	1.410 (1.246-3682)	0.001^∗^	0.386 (0.094-1.590)	0.181
Marital status (unmarried vs. married)	3.007 (1.901-4.756)	<0.001^∗^	4.659 (2.344-6.466)	<0.001^∗^
Smoking (yes vs. no)	1.433 (1.267-3.702)	0.001^∗^	1.029 (1.007-2.114)	<0.001^∗^
Usage of mechanical ventilator (yes vs. no)	1.444 (1.084-2.245)	0.001^∗^	1.216 (1.105-2.447)	<0.001^∗^
Head trauma (yes vs. no)	1.651 (1.054-3.150)	<0.001^∗^	1.403 (1.170-2.954)	0.039^∗^
APACHE IV score	1.269 (1.191-1.352)	<0.001^∗^	1.122 (0.964-1.305)	0.138
SOFA score	2.338 (1.963-2.784)	<0.001^∗^	1.435 (1.240-2.788)	0.006^∗^
RASS score	1.020 (0.852-1.356)	0.993		
VAP (yes vs. no)	0.403 (0.208-0.781)	0.007^∗^	0.427 (0.218-0.835)	0.053
Hospital LOS	1.623 (1.484-1.747)	<0.001^∗^	1.121 (0.926-1.357)	0.240
ICU LOS	3.486 (2.505-4.852)	<0.001^∗^	3.904 (2.464-6.183)	<0.001^∗^

APACHE IV: Acute Physiology and Chronic Health Evaluation IV; SOFA: Sequential Organ Failure Assessment; RASS: Richmond Agitation-Sedation Scale; MV: mechanical ventilator; VAP: ventilator-associated pneumonia; LOS: length of stay; OR: odds ratio. ^∗^Statistically significant <0.05.

**Table 3:** Proportional hazard Cox regression analysis to determine the independent factors associated with delirium.

Variables	Univariate		Multivariate	
	HR (95% CI)	*P* value	HR (95% CI)	*P* value
Age	1.006 (0.991-1.021)	0.424		
Gender (male vs. female)	0.689 (0.429-1.106)	0.123		
Marital status (unmarried vs. married)	0.990 (0.667-1.447)	0.958		
Smoking (yes vs. no)	1.850 (1.145-2.988)	0.012^∗^	0.869 (0.396-1.910)	0.727
Usage of mechanical ventilator (yes vs. no)	1.417 (1.237-3.735)	0.002^∗^	1.523 (1.197-2.388)	0.001^∗^
Head trauma (yes vs. no)	1.517 (1.014-2.267)	0.042^∗^	1.312 (0.837-2.059)	0.237
APACHE IV score	1.034 (0.982-1.090)	0.207		
SOFA score	0.932 (0.803-1.082)	0.355		
RASS score	1.217 (0.940-1.576)	0.135		
VAP (yes vs. no)	0.458 (0.259-0.811)	0.007^∗^	0.488 (0.274-0.871)	0.055
Hospital LOS	0.975 (0.933-1.019)	0.258		
ICU LOS	0.969 (0.913-1.028)	0.291		

APACHE IV: Acute Physiology and Chronic Health Evaluation IV; SOFA: Sequential Organ Failure Assessment; RASS: Richmond Agitation-Sedation Scale; MV: mechanical ventilator; VAP: ventilator-associated pneumonia; LOS: length of stay; HR: hazard ratio. ^∗^Statistically significant <0.05.

**Table 4:** Cumulative doses of sedative and analgesic medications in patients with and without delirium.

Drugs	Patients without delirium (*n* = 292)		Patients with delirium (*n* = 108)		*P*-value
	No.	Mean ± SD	No.	Mean ± SD	
Morphine	6	5 (mg)	108	46.85 ± 23.33 (mg)	<0.001
Methadone	268	12.27 ± 8.78 (mg)	108	88.84 ± 18.56 (mg)	<0.001
Fentanyl	116	382.75 ± 64.97 (*μ*g)	86	2837.20 ± 353.81 (*μ*g)	<0.001
Midazolam	0	—	108	86.34 ± 69.17 (mg)	—
Dexamethasone	48	62.50 ± 20.90 (mg)	80	84.80 ± 20.50 (mg)	<0.001
Diazepam	0	—	108	86.34 ± 69.17 (mg)	—
Hydrocortisone	16	300 (mg)	0	—	—

## Data Availability

Data are available from the first and corresponding authors upon a reasonable request.

## Abbreviations

ICUs: Intensive care units
LOS: Hospital length of stay
OR: Odds ratio
HR: Hazard ratio
CIs: Confidence intervals
CAM-ICU: Confusion Assessment Method for the ICU
ICDSC: Intensive Care Delirium Screening Checklist
APACHE IV score: Acute Physiology and Chronic Health Evaluation IV
SOFA score: Sequential Organ Failure Assessment
RSS: Ramsay Sedation Scale
RASS: Richmond Agitation Sedation Scale
MV: Mechanical ventilation
VAP: Ventilator-associated pneumonia.

## Supplementary Materials

Supplementary Materials Supplementary Table 1: time trend of severity illness scoring systems in ICU patients with and without delirium.
