# Supplementary material for: Incidence and Risk Factors of Delirium in the Intensive Care Unit: A Prospective Cohort
Source: Biomed Res Int. 2021 Jan 8;2021:6219678. doi: 10.1155/2021/6219678 (PMC7810554; doi:10.1155/2021/6219678)
Supplement: Supplementary Materials — Supplementary Table 1: time trend of severity illness scoring systems in ICU patients with and without delirium. [file 6219678.f1.docx]

**Supplementary Table 1: Time trend of severity illness scoring systems in ICU patients with and without delirium**

| **Scoring system** | **Delirium** | **Time Trend** | | | | | **P-**  **value**** | **P-**  **value***** |
| --- | --- | --- | --- | --- | --- | --- | --- | --- |
|  |  | **Day 1** | **Day 2** | **Day 3** | **Day 4** | **Day 5** |  |  |
| **APACHE IV** | No | 11.57±4.11 | 9.13±5.02 | 8.25±5.41 | 7.28±5.34 | 7.85±6.64 | <0.001 | 0.001 |
|  | Yes | 16.08±3.84 | 15.25±5.02 | 15.14±5.70 | 14.02±6.77 | 13.08±7.50 | <0.001 |  |
| ***P-value** | | <0.001 | <0.001 | <0.001 | <0.001 | <0.001 |  |  |
| **SOFA** | No | 4.93±1.70 | 4.02±2.21 | 3.55±2.54 | 3.02±2.60 | 3.46±3.23 | <0.001 | 0.002 |
|  | Yes | 7.37±1.17 | 7.49±0.96 | 6.27±1.23 | 5.82±1.56 | 5.77±1.85 | <0.001 |  |
| ***P-value** | | <0.001 | <0.001 | <0.001 | <0.001 | <0.001 |  |  |
| **RASS** | No | 1.48±0.50 | 1.59±0.83 | 0.78±0.83 | 0.78±1.07 | 0.37±1.26 | <0.001 | <0.001 |
|  | Yes | 2.83±0.81 | 3.25±0.44 | 3.18±0.75 | 3.74±0.44 | 3.50±0.74 | <0.001 |  |
| ***P-value** | | <0.001 | <0.001 | <0.001 | <0.001 | <0.001 |  |  |
| **Scoring system** | **Delirium** | **Time Trend** | | | | | **P-**  **value**** | **P-**  **value***** |
|  |  | **Day 6** | **Day 7** | **Day 8** | **Day 9** | **Day 10** |  |  |
| **APACHE IV** | No | 9.06±8.80 | 9.54±9.82 | 15.32±13.84 | 12.90±13.69 | 10.57±12.58 |  |  |
|  | Yes | 13.09±7.66 | 13.09±7.66 | 11.33±8.89 | 10.96±8.90 | 10.29±9.74 |  |  |
| ***P-value** | | <0.001 | 0.021 | 0.042 | 0.346 | 0.901 |  |  |
| **SOFA** | No | 4.38±4.28 | 4.51±4.63 | 7.71±5.41 | 5.82±5.02 | 4.96±4.54 |  |  |
|  | Yes | 5.70±1.86 | 5.11±2.24 | 4.92±3.26 | 4.49±3.26 | 4.53±3.36 |  |  |
| ***P-value** | | 0.003 | 0.229 | <0.001 | 0.088 | 0.597 |  |  |
| **RASS** | No | -0.77±2.24 | -1.31±2.43 | -2.57±2.85 | - | - |  |  |
|  | Yes | 3.25±1.11 | 3.01±0.72 | 2.77±0.63 | 1.91±0.76 | 1.65±0.47 |  |  |
| ***P-value** | | <0.001 | <0.001 | <0.001 | - | - |  |  |
| **Scoring system** | **Delirium** | **Time Trend** | | | | | **P-**  **value**** | **P-**  **value***** |
|  |  | **Day 11** | **Day 12** | **Day 13** | **Day 14** | **Day 28** |  |  |
| **APACHE IV** | No | 31.20±13.91 | 35.50±4.94 | 35 | - | - |  |  |
|  | Yes | 10.05±10.19 | 9.20±9.51 | 8.57±9.04 | 7.38±7.74 | 28 |  |  |
| ***P-value** | | <0.001 | <0.001 | <0.001 | - | - |  |  |
| **SOFA** | No | 12.25±5.50 | 15 | 15 | 15 | - |  |  |
|  | Yes | 4.05±3.45 | 4.01±3.15 | 4.27±3.12 | 3.94±2.62 | 9 |  |  |
| ***P-value** | | <0.001 | <0.001 | <0.001 | <0.001 | - |  |  |
| **RASS** | No | - | - | - | - | - |  |  |
|  | Yes | 0.53±0.50 | 0.87±0.64 | 1.26±0.44 | 1 | - |  |  |
| ***P-value** | | - | - | - | - | - |  |  |

Abbreviation; APACHE IV: Acute Physiology and Chronic Health Evaluation IV; SOFA: Sequential Organ Failure Assessment; RASS: Richmond Agitation-Sedation Scale * Independent t-test between two groups, ** Time-interaction within group based on RMANOVA, *** Time-interaction between two groups
